# Identification of multiple binding sites for the THAP domain of the *Galileo* transposase in the long terminal inverted-repeats^☆^

**DOI:** 10.1016/j.gene.2013.04.050

**Published:** 2013-08-01

**Authors:** Mar Marzo, Danxu Liu, Alfredo Ruiz, Ronald Chalmers

**Affiliations:** a School of Biomedical Sciences, University of Nottingham, Queen's Medical Centre, Nottingham NG7 2UH, UK; b Departament de Genètica i Microbiologia, Universitat Autònoma de Barcelona, 08193 Bellaterra, Barcelona, Spain

**Keywords:** TIR, terminal inverted repeat, bp, base pair, kb, kilobase, MBP-tag, maltose binding protein expression tag, EMSA, electrophoretic mobility shift assay, ORF, open reading frame, BS, binding site, Dbuz, *Drosophila buzzatii*, Dmoj, *Drosophila mojavensis*, Dana, *Drosophila ananassae*, *P-element*, *Foldback*, THAP domain, DNA binding, Evolution

## Abstract

*Galileo* is a DNA transposon responsible for the generation of several chromosomal inversions in *Drosophila*. In contrast to other members of the *P-element* superfamily, it has unusually long terminal inverted-repeats (TIRs) that resemble those of *Foldback* elements. To investigate the function of the long TIRs we derived consensus and ancestral sequences for the *Galileo* transposase in three species of Drosophilids. Following gene synthesis, we expressed and purified their constituent THAP domains and tested their binding activity towards the respective *Galileo* TIRs. DNase I footprinting located the most proximal DNA binding site about 70 bp from the transposon end. Using this sequence we identified further binding sites in the tandem repeats that are found within the long TIRs. This suggests that the synaptic complex between *Galileo* ends may be a complicated structure containing higher-order multimers of the transposase. We also attempted to reconstitute *Galileo* transposition in *Drosophila* embryos but no events were detected. Thus, although the limited numbers of *Galileo* copies in each genome were sufficient to provide functional consensus sequences for the THAP domains, they do not specify a fully active transposase. Since the THAP recognition sequence is short, and will occur many times in a large genome, it seems likely that the multiple binding sites within the long, internally repetitive, TIRs of *Galileo* and other *Foldback*-like elements may provide the transposase with its binding specificity.

## Highlights

• Long TIR is a trait found in different superfamilies of DNA transposons.
• Long TIR increases the length of the transposon decreasing transposition efficiency.
• We have reconstructed functional protein sequences of the long-TIR *Galileo* element.
• Multiple transposase binding sites have been found in the long *Galileo* TIR.
• Long TIR multiple binding sites may offset the negative effect of transposon length.

## Graphical abstract

**Figure:**
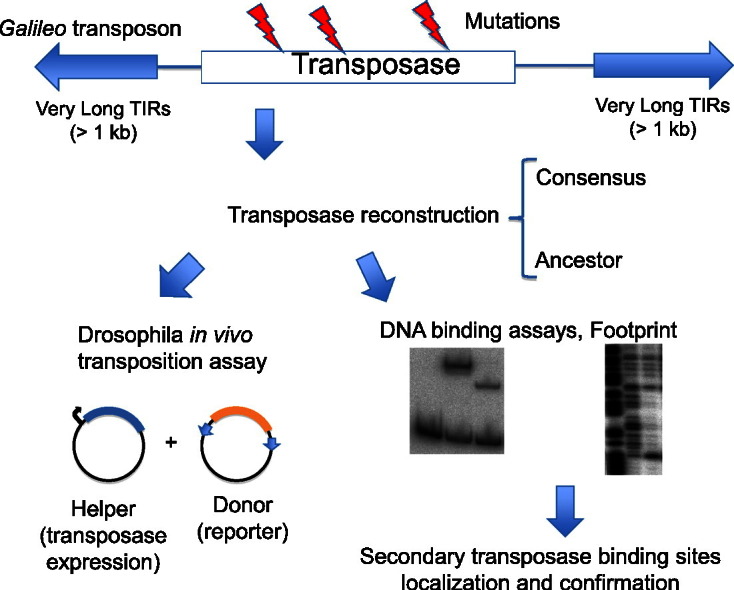

## 1 Introduction

Transposable elements (TEs) are mobile genetic components of virtually all eukaryotic species (Feschotte and Pritham, 2007; Wicker et al., 2007). These repetitive sequences make up a substantial proportion of most genomes and have a huge impact on the evolution of their hosts (Adams et al., 2000; Feschotte and Pritham, 2006; Jurka et al., 2007; Lander et al., 2001; Morgante, 2006). TEs are diverse and employ many different mechanisms for mobilization. Two major groups of transposons are recognized depending on whether they have an RNA intermediate or a DNA intermediate (Finnegan, 1989). Transposons are then further classified into numerous superfamilies and families depending on their sequence structure and similarity (Feschotte and Pritham, 2007; Jurka et al., 2007; Wicker et al., 2007).

All TE families contain autonomous and non-autonomous members. Autonomous transposons retain the ability to express the protein(s) required for their own transposition. Non-autonomous copies contain indels or point mutations that render them non-functional. The non-autonomous copies exploit the gene products of the autonomous copies, which they often outnumber (Feschotte and Pritham, 2007).

Biochemical analysis of transposition reactions helps us to understand how the elements behave in the genome, and allows the development of transposons as genetic tools. Since most of the transposon copies in higher eukaryotic genomes harbor mutations in their coding regions, different strategies have been used to reconstitute their activity. Sometimes, a simple consensus sequence constructed from different copies results in the restoration of activity e.g. *Himar1*, *Frog Prince* and *Harbinger* transposases (Kapitonov and Jurka, 2004; Lipkow et al., 2004; Miskey et al., 2003). Often, the amplification of non-autonomous transposons means that a simple consensus sequence encodes a non-functional transposase protein. In such cases the functional ancestral sequence may be reconstructed by taking account of phylogenetic information while building the consensus. For example, this approach has been used for the revival of *Hsmar1* (Miskey et al., 2007).

The *P-element* was discovered in *Drosophila melanogaster* as the agent responsible for P-M hybrid dysgenesis (Kidwell, 1985; Rubin et al., 1982). It has since been studied *in vivo* and *in vitro* and is now widely used as a tool for genomic analysis of *D. melanogaster* (Rio, 2002; Ryder and Russell, 2003). The *P-element* defines a superfamily of DNA transposons, which includes *1360* and *Galileo* (see below). These elements harbor a transposase coding region flanked by TIRs, which are needed for the transposition reaction. The *P-element* transposase contains four functional domains: an N-terminal DNA binding domain, a coiled coil region presumably involved in protein–protein interactions, a GTP binding domain and a catalytic domain with four key acidic residues, which may coordinate the catalytic metal ions (Rio, 2002; Sabogal and Rio, 2010). The *P-element* catalytic domain is thought to belong to the RNase H-like superfamily of polynucleotidyl transferases (Hickman et al., 2010; Rio, 2002; Sabogal and Rio, 2010; Yuan and Wessler, 2011).

The *P-element* transposase contains a THAP domain, which is presumably involved in site-specific DNA binding. The THAP domain is an evolutionary conserved motif shared by different animal proteins, including cell-cycle regulators, pro-apoptotic factors, transcriptional repressors and chromatin-associated proteins (Clouaire et al., 2005; Quesneville et al., 2005; Roussigne et al., 2003). The domain has a long zinc finger (~ 90 amino acids) in which key residues are highly conserved (Roussigne et al., 2003). Crystal structures have been reported for the human THAP1 protein and the *D. melanogaster P-element* transposase (Campagne et al., 2010; Sabogal et al., 2010). These show that the THAP domain interacts with its binding sequence in a bipartite manner, through the major and minor grooves of the DNA.

**Fig. 1:**
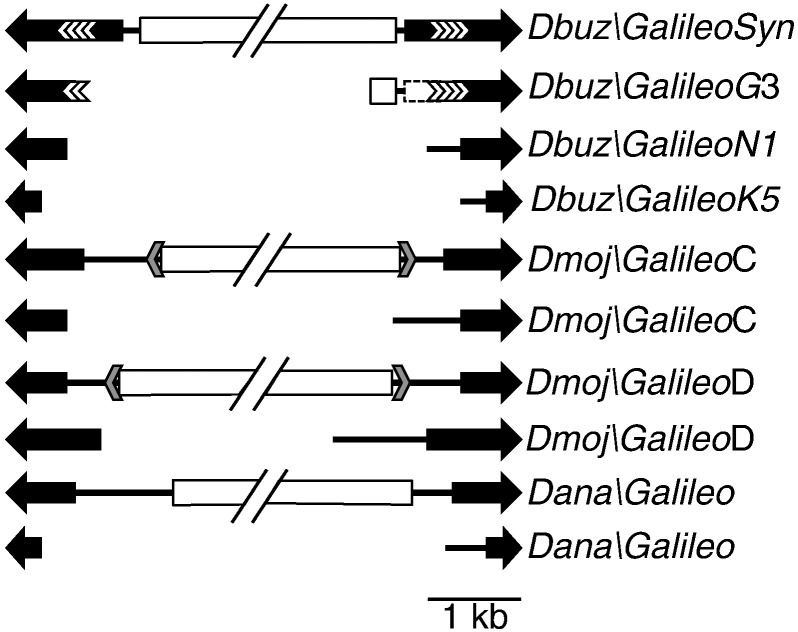
Structure of representative *Galileo* copies in the different species of *Drosophila* used in this work. The black arrows are the TIR and white chevrons are tandem repeats within the respective TIRs. The white rectangles are the transposase coding regions. None of the transposase-containing copies harbor a functional ORF. The grey arrowheads are internal inverted repeats found in the *D. mojavensis* examples.

The *Galileo* transposon was discovered in *Drosophila buzzatii*, where it has caused three large chromosomal inversions, which are currently segregating naturally in the population (Cáceres et al., 1999; Casals et al., 2003; Delprat et al., 2009). Although originally considered a *Foldback*-like element, it was later included in the *P-element* superfamily of cut-and-paste transposons based on the sequence of the putative transposase (Marzo et al., 2008). *Galileo* is probably widespread within the *Drosophila* genus because it has been found in species of the two main subgenera, *Sophophora* and *Drosophila* (Marzo et al., 2008). Many incomplete (non-autonomous) copies of *Galileo* have been detected in all species searched and in some cases two or more *Galileo* subfamilies have been found coexisting in the same genome (Fig. 1). For instance, three subfamilies are present in *D. buzzatii*, while *Drosophila mojavensis* harbors four subfamilies (Delprat et al., 2009; Marzo et al., 2008). To date all sequenced copies of the transposon harbor premature stop codons and/or frame-shift mutations. Nevertheless, the sequence remnants reveal that the main domains of the *P-element* transposase are present in *Galileo*.

The most conspicuous features of *Galileo* are the TIRs which are 0.5 to 1.2 kb in length. This is considerably longer than other members of the *P-element* superfamily, in which the TIRs range from 30 to 50 bp. Indeed, it was the extreme length of *Galileo* TIRs that defined it as a *Foldback*-like transposon before it was recognized as a member of the *P-element* superfamily. The *Galileo* TIRs have another interesting property: namely, that the sequence conservation between elements in different species is restricted to ~ 40 bp at the extremities of the transposon (Marzo et al., 2008). One obvious possibility is that these regions are functional transposition sequences, and would be the equivalent to the short TIRs of the *P-element*. If true, this leaves the function of the remaining 0.5 to 1.2 kb open to question. The fact that they are not conserved between elements in different species, and that they contain internal tandem repeats in some subfamilies, has led to the suggestion that structure of the DNA may play a role in transposition (Adams et al., 2000; Ivics et al., 1997; Marquez and Pritham, 2010; Moschetti et al., 2008). The mechanism of *Galileo* transposition may therefore prove to be of considerable interest, and may explain the frequency with which this element is able to generate chromosomal inversions in *Drosophila*. In the present work we have focused on the reconstruction of an active transposase and its binding to the TIR. Although we have not succeeded in a full reconstitution of the transposition reaction, we have detected transposase binding to the extremities of *Galileo* and identified secondary binding sites in the tandem repeats of the TIR. This represents the first steps in the characterization of *Galileo* recombination. Further characterization promises to reveal fascinating details of the interactions between this transposon and its host and perhaps even the reason it promotes chromosomal inversions so frequently.

## 2 Results

### 2.1 *Galileo* transposase sequence reconstruction

The most complete example of the *Galileo* transposon is from *D. buzzatii* and was reconstructed from four overlapping PCR products (Marzo et al., 2008). Following the convention for *Drosophila* transposons (www.flybase.org) we will refer to this element as Dbuz\*Galileo*, with the suffix ‘Syn’ (from synthetic) to indicate that it is a conceptual putatively complete copy (Fig. 1). This element has TIRs of 1.2 kb and an intron-less ORF encoding a 912 amino acid transposase (after correcting two stop codons and a frame shift mutation). Although there is no complete genome sequence for *D. buzzatii*, several internally deleted *Galileo* elements have been identified at the junctions of chromosomal rearrangements, and in other PCR and library screening experiments (Cáceres et al., 2001; Casals et al., 2005). Some of these elements were originally called *Kepler* and *Newton* but later assigned to different subfamilies of *Galileo*, now known as Dbuz\*Galileo*-K and Dbuz\*Galileo*-N, while Dbuz\*Galileo*-G denotes the subfamily of the synthetic element. The various *Galileo* subfamilies have TIRs of different lengths, but share significant sequence homologies at the tips of the elements where one might expect the transposase to bind (~ 50 bp). Three specific examples of internally deleted G, N and K subfamily members are shown in Fig. 1. The complete genome sequences for *Drosophila ananassae* and *D. mojavensis* contained additional *Galileo* elements. In *D. ananassae* there is a single *Galileo* subfamily designated Dana\*Galileo*. In *D. mojavensis* there are four subfamilies, two of which harbored transposase sequences: Dmoj\*Galileo*-C and Dmoj\*Galileo*-D (Marzo et al., 2008). These transposons all contain internal deletions, and two examples of members of each subfamily are shown in Fig. 1.

We were most interested in the Dbuz\*Galileo*-G elements because these provide a complete transposase and have probably caused two of the three natural chromosomal deletions (Marzo et al., 2008). To recover further examples, in addition to the single synthetic element, we used PCR to amplify three overlapping segments from eight strains of *D. buzzatii*. Analysis of the products yielded a 2958 bp assembly of DNA sequences, which was used to generate a consensus by the majority rule. The consensus, designated Dbuz\*Galileo*-Consensus, was five nucleotides different from the synthetic copy and encoded a full-length transposase without stop codons or frame shifts.

### 2.2 *Galileo in vivo* transposition

To test whether the Dbuz/*Galileo*-Consensus transposase was capable of supporting transposition in *Drosophila* embryos, we adapted the *P-element* general-transformation system (Rubin and Spradling, 1982). The system consists of two plasmids, which are co-injected into *white* (*w-*) *Drosophila* embryos: a helper plasmid, which encodes the *P-element* transposase under the control of a heat shock promoter; and a reporter plasmid, which encodes a *P-element* with a mini-white reporter gene. Transposition yields transgenic animals, which can be scored for the wild type red eye color after back crossing to the original *w-* strain. We adapted this system by replacing the *P-element* transposon ends with *Galileo* ends, and replacing the *P-element* transposase with the Dbuz/*Galileo*-Consensus transposase.

We performed three sets of embryo injections (Table 1). The first was a positive control using the unmodified *P-element* plasmids. In the second set the *Galileo* helper and reporter plasmids were injected. The third set was a negative control in which the *Galileo* reporter plasmid was injected alone. In the *P-element* experiment 19 of the 91 crosses yielded a total of 384 F1s with red eyes. Neither of the *Galileo* experiments yielded any transgenic animals. Furthermore, none of the F0 animals, which developed from the injected embryos, showed any indication of eye mosaicism.

### 2.3 THAP domain sequence reconstruction

**Fig. 2:**
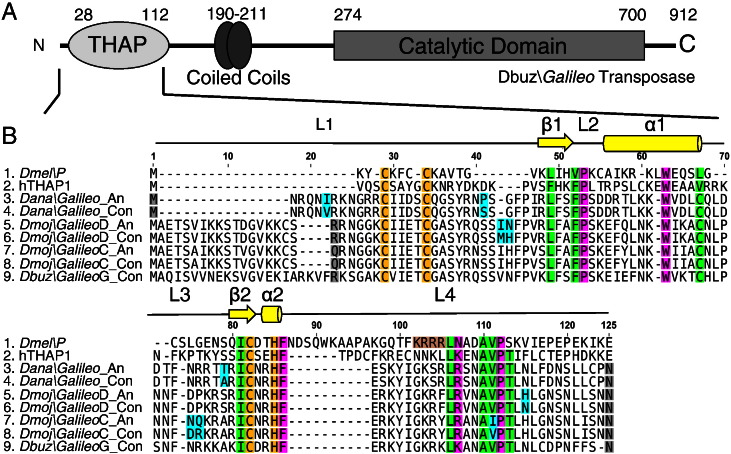
THAP domain protein sequences. A) Domain structure of the predicted *Galileo* transposase: the THAP is a DNA binding domain, the coiled coil region is probably responsible of protein–protein interactions (represented as two overlapping circles) and the catalytic domain is in the C-terminal region. B) Alignment of the consensus and ancestral *Galileo* THAP domain sequences with the THAP domain of the *P-element* transposase (*D. melanogaster*) and THAP1 protein (*Homo sapiens*). The predicted secondary structures are shown above the alignment (adapted from Bessière et al., 2008; Sabogal et al., 2010). Yellow arrows represent β sheets and yellow cylinders are α helical regions. Key residues are colored: yellow: zinc coordination residues (C2CH), green: conserved hydrophobic residues, pink: invariant residues, light brown: nuclear localization signal (NLS) of the *P-element* transposase. The residues cloned for protein expression are those between the grey shaded ones. The residues colored in cyan are the amino acid changes between ancestor and consensus sequences.

*In silico* analysis of the synthetic Dbuz\*Galileo*-G transposase previously revealed a putative THAP DNA-binding domain near the N-terminus of the protein (Marzo et al., 2008) (Fig. 2A). We therefore set out to discover its specific binding site within the transposon and the extent of cross-reactivity between the various *Galileo* subfamilies. The first 125 amino acids of the transposase were used as a query to search the genome sequences of *D. ananassae* and *D. mojavensis*. The majority rule consensus sequences generated for the three respective genomes encoded a protein with the key features of a functional THAP domain (Fig. 2B).

To avoid artifacts arising from the historical amplification of non-autonomous copies, we used the maximum likelihood method, which takes account of the phylogeny of sequences to infer the ancestral states of the respective THAP domains. The ancestral sequences each had two to three amino acid differences from the respective majority-rule consensus sequences (Fig. 2B). Only one of these differences, at position 111, was in a highly-conserved region. However, this was a conservative valine to isoleucine substitution.

Alignment of the *Galileo* sequences with the *P-element* THAP domain and the human THAP1 protein revealed several indels (Fig. 2B). The most significant difference is that the *Galileo* THAP domains have an extended N-terminus. This may be functionally significant because the initial methionine in the *P-element* THAP domain interacts directly with the DNA binding site. Loop-4 in the *Galileo* THAP domains is shorter than in the other THAP domains (L4 in Fig. 2B). In the crystal structure of the *P-element* DNA binding domain, this loop contributes to DNA binding specificity by making minor groove contacts (Sabogal et al., 2010). The zinc finger CCCH motif, which coordinates the metal ion, is conserved in all of the *Galileo* THAP domains.

### 2.4 THAP domains bind the cognate TIRs

**Fig. 3:**
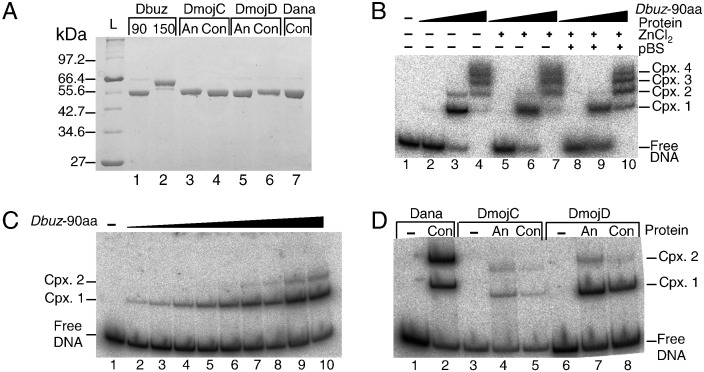
Protein expression, purification and DNA binding. A) SDS-PAGE with the 7 expressed THAP domain proteins, ~ 5 μg of each protein was loaded as indicated. *D. buzzatii* protein was either 90 or 150 amino acids from the N-terminus of the transposase. The others were 90 amino acids long. B) EMSA performed with Dbuz\*Galileo*-THAP-90aa. Three different binding conditions were tested. First lane is Dbuz\*Galileo*G labeled TIR (2.2 nM). Lanes 2, 3 and 4 are × 100 increasing protein concentrations (470 pM, 47 nM and 4.7 μM). Lanes 5, 6 and 7 are the same but with 100 μM ZnCl_2_. Lanes 8, 9 and 10 are the same but with 500 ng of pBluescript as competitor. Note that the proteins were purified in a buffer containing EDTA and reactions in which zinc was not added back contained only that zinc acquired by the proteins during folding. C) Fine titration EMSA of the Dbuz\*Galileo*-THAP-90aa with its TIR (0.14 nM). Protein concentration increases 2-fold in successive lanes: 0.184 nM, 0.367 nM, 0.734 nM, 1.469 nM, 2.938 nM, 5.875 nM, 11.75 nM, 23.5 nM, 47 nM and 94 nM. D) EMSA in which binding of the indicated 90 amino acid THAP domains is tested against the consensus TIR of their respective *Galileo* sub-group. Final protein concentration: 5.8 nM and TIR final concentration is 0.28 nM. Note that it is not necessary to reconstruct the ancestral TIR because, unlike a transposase gene, a non-functional TIR can not be amplified by transposition. The consensus TIR can therefore be expected to retain functionality.

The consensus and ancestral sequences of the various THAP domains were codon optimized for expression in *Escherichia coli* and chemically synthesized. These sequences were fused to the maltose binding protein gene (MBP), which was used as an affinity purification tag (Fig. 3A). Only six of the THAP domains were purified because we were unable to clone the Dana/*Galileo* ancestral sequence into the expression vector. The structure of the *P-element* THAP domain suggested that the first 90 amino acids would provide an independently folding domain. We also purified the 150 amino acid N-terminal domain of the Dbuz/*Galileo*-G consensus domain.

Our experiments focused first on the properties of the 90 amino acid domain from the *D. buzzatii* consensus protein. We used an electrophoretic mobility shift assay (EMSA) to test its binding to the cognate TIR (Fig. 3B). Titration of the protein concentration revealed four retarded complexes. In addition to the primary shift (Complex 1), three super-shifted bands were detected (indicated as Cpx. 2, 3 and 4). We will present evidence below that the super-shifted bands are probably caused by multimerization of the protein, rather than by the presence of multiple binding sites in the TIR. The four complexes were not affected by the presence of pBluescript, which was added as a non-specific competitor DNA. Once properly folded, zinc finger proteins, such as the THAP domain, bind the metal ion very tightly and exchange with the bulk phase is generally slow or absent. They are therefore often insensitive to chelating agents. Although the *D. buzzatti* THAP domain was purified in the presence of a chelating agent, it retained DNA binding activity, which was not enhanced by the addition of zinc chloride (Fig. 3B). It therefore seems that the protein probably retains its metal ion throughout the purification procedure.

To further investigate the properties of the super-shifted bands we performed a fine titration with the *D. buzzatii* consensus protein (Fig. 3C). There was a clear progression in which Complex 2 became prominent just as Complex 1 was reaching completion. The concentration of the labeled TIR in these experiments was less than 10% of that shown in Fig. 3B. Nevertheless, the pattern of retardation was almost identical in each experiment when the THAP protein concentration was 47 nM (i.e. in lanes 3 and 9 in parts B and C, respectively). This suggests that under these conditions, binding is determined by the absolute concentrations of the binding partners and not by the ratio of transposase to transposon ends, as is observed in some systems.

Binding of the remaining five THAP domains to their cognate TIRs is shown in Fig. 3D. At the intermediate protein concentration used, all five domains produced the first two retarded bands detected in the Dbuz\*Galileo*-G titrations. The *D. mojavensis* and *D. ananassae* ancestral proteins appeared to bind to the respective TIRs slightly better than the corresponding consensus sequences. However, the differences are probably not significant and are within the normal range of variation of an EMSA. We also tested the sensitivity of the *D. mojavensis* and *D. ananassae* domains to the presence of zinc and competitor DNA in the binding reaction. Both behaved similarly to the *D. buzzatii* domain and were unaffected by these reagents (not shown).

### 2.5 Cross-reactivity between the subfamilies

**Fig. 4:**
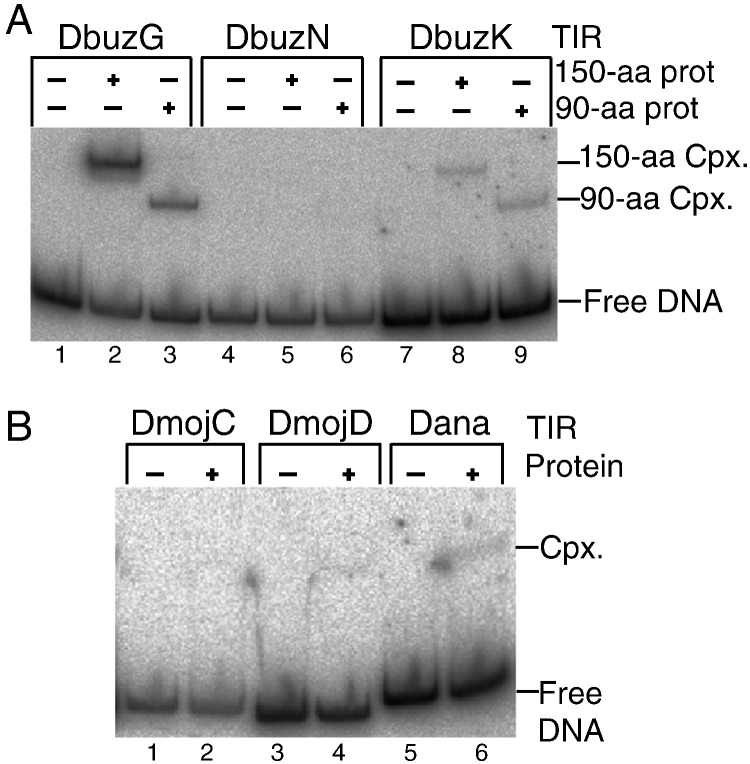
Cross-binding EMSA experiments. A) The 90 and 150 amino acid purified THAP domain proteins from *D. buzzatii* were tested for binding to the consensus TIRs from the indicated subfamilies. Final protein concentration and TIR concentration are 5.8 nM and 0.28 nM. B) Dbuz\*Galileo*-THAP-90aa against Dmoj\*Galileo*C-TIR (lane 2), Dmoj\*Galileo*D-TIR (lane 4), Dana\*Galileo* TIR (lane 6).

Since the *D. buzzatii* genome harbors G, K and N subfamilies of *Galileo*, we wondered about the extent of cross-reactivity between the transposase and the various TIRs. We therefore used an EMSA to test the ability of the G subfamily consensus THAP domain to bind the N and K subfamily TIRs (Fig. 4A). This revealed cross-reactivity with the K subfamily TIR, which was significantly weaker than binding to the cognate TIR. This protein was unable to bind the N subfamily TIR (Fig. 4A). We next tested whether the G subfamily consensus THAP domain could bind the TIRs from the *Galileo* subfamilies C and D from *D. mojavensis* and the single representative from *D. ananassae* (Fig. 4B). There was significant binding towards the *D. ananassae* TIR (lane 6). We also detected cross-reactivity with the *D. mojavensis* D element (lane 4), but it is so slight that it probably lacks biological significance.

### 2.6 Identification of the THAP domain binding site

**Fig. 5:**
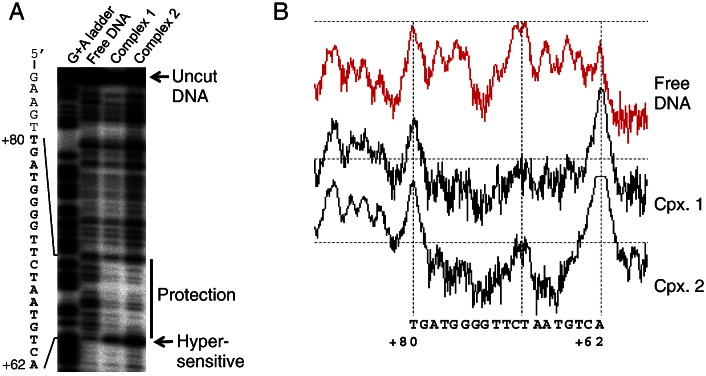
Identification of the proximal THAP binding site. A) Complexes were formed using the G subfamily 90 amino acid consensus THAP domain and the 150 bp consensus TIR, which was radiolabeled (see Fig. 3B, lane 3 for an example). The complexes were footprinted with DNaseI and resolved on a DNA sequencing gel. The radioactive signals were recorded on a phosphoimager. The protected DNA sequence was shown on the left of the gel. The entire image of the gel is shown uncropped in Fig. S1. A) Complexes were formed using the G subfamily 90 amino acid consensus THAP domain and the 150 bp consensus TIR, which was radiolabeled (see Fig. 3B, lane 3 for an example). The complexes were footprinted with DNaseI and resolved on a DNA sequencing gel. The radioactive signals were recorded on a phosphoimager. The protected DNA sequence was shown on the left of the gel. The entire image of the gel is shown uncropped in Fig. S1. B) Densitometric traces of the gel in part A were made using the Fuji Image Gauge phosphoimager software. Traces were exported as PICT images and combined in the Apple Works vector drawing program.

We next used DNase I footprinting to locate the binding site of the G subfamily consensus THAP domain within the cognate TIR (Fig. 5). The protein was mixed with radiolabeled TIR, treated with DNase I and the mixture was resolved using the EMSA. Complexes 1 and 2 were recovered from the gel and the footprint was displayed on a DNA sequencing gel. There was a single protected region of 18 bp spanning position + 63 to + 80 of the 150 bp TIR fragment. There was also a hypersensitive position at the end of the protected region. Complexes 1 and 2 produced the same protection pattern, suggesting that the super-shift experienced by Complex 2 may be due to the oligomeric state of the transposase, rather than the binding of additional monomers to the DNA. It seems unlikely that the super-shifted complexes are due to non-specific DNA binding because they are unaffected by the presence of non-specific competitor DNA (Fig. 3B). It should be noted that the protected region lies outside of the ~ 40 bp terminal sequence conserved between the different families of *Galileo* elements. As expected from the cross-reactivity experiments in Fig. 4, no protection was detected in this region. This can be seen in the image of the entire gel presented in Fig. S1.

**Fig. 6:**
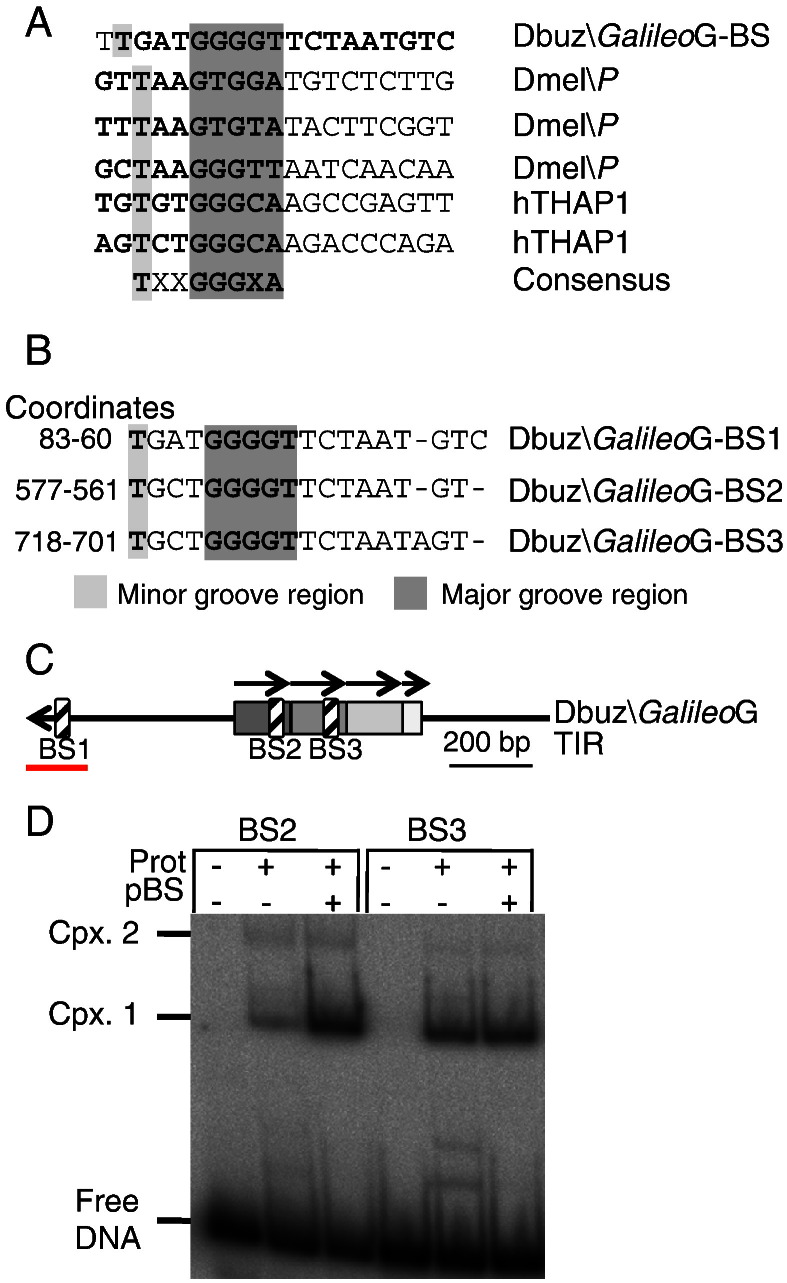
THAP domain binding sequence comparison. A) Dbuz\*Galileo*G compared to *Dmel\P-element* and hTHAP1 binding sites (Bessière et al., 2008; Campagne et al., 2010; Sabogal et al., 2010). The major and minor groove interacting regions are colored. A putative consensus THAP binding sequences, including Dbuz\*Galileo*G sequences is deduced. B) Alignment of the Dbuz\*Galileo*G binding site with other putative binding sites found downstream in the Dbuz\*Galileo*G-TIR. C) Structure of the Dbuz\*Galileo*G-TIR where the tandem repeats are drawn as grey rectangles and the binding sites are drawn with hatched shading (BS1, BS2 and BS3). The red bar depicts the 150 bp TIR consensus region used in the different experiments. A 200 bp scale bar is also provided. D) The putative secondary binding sites in *Galileo* are functional. 50 bp oligonucleotides encoding the putative *Galileo* secondary binding sites identified in part C were tested for binding in an EMSA using the Dbuz\*Galileo*G-90aa DNA binding domain (47 nM). pBluescript (500 ng) was added as an non-specific competitor. The location of BS2 and BS3 is shown in part C.

DNase footprints extend further than the actual protein binding site because of steric hindrance. To identify the core sequence, we aligned the *Galileo* THAP binding site with the *P-element* THAP sites, together with two further well-defined examples in humans (Fig. 6A). This revealed a conserved region towards the 5′ end of the protected region. We searched for this sequence in the K and N subfamily members, and in the *D. mojavensis* and *D. ananassae Galileo* elements, but no significant matches were obtained. However, when we searched within *Galileo* G itself, we found two additional highly significant matches (BS2 and BS3 in Fig. 6B). These putative binding sites are located within the first two of the four long direct-repeats that comprise part of the 1.2 kb TIR of *Galileo*-G (Fig. 6C). The THAP binding site appeared to be absent from the third and fourth repeats. Finally, we used the EMSA to show that the Dbuz\*Galileo* THAP domain binds to BS2 and BS3, as would be expected from the high degree of sequence conservation, particularly in the core region (Fig. 6D).

## 3 Discussion

Since no fully functional *Galileo* sequences have been identified to date, we constructed consensus ORFs from a limited number of copies. Although no transposition was detected in an *in vivo* assay, we were able to demonstrate DNA binding by the N-terminal THAP domains of various members of the transposase family. We examined the *D. buzzatii Galileo*-G transposon in most detail, and identified the precise location of the THAP binding site, centered about 70 bp from the transposon end (Fig. 5). This lies outside the ~ 40 bp terminal segments conserved between different *Galileo* families, but within the family-specific long TIRs. Two additional THAP binding sites were identified at 561–577 bp and 701–718 bp of the transposon ends (Fig. 6). The first binding site occupies a location similar to the THAP binding site of the *P-element*, which is located at bp 53–63 in the 5′ end and bp 41–51 in the 3′ end the transposon (Rio, 2002). It therefore seems unlikely that the THAP DNA binding domain plays a direct role in catalysis in the *P-element* or *Galileo*. Rather, it probably increases the specificity of transposon end recognition by providing the transposase with secondary binding determinants.

The existence of secondary binding sites, or transposition enhancers, has been reported in different transposons. These sequences may or may not be part of the TIR. For example, *P-element* has subterminal transposition enhancers located outside the short TIR, whereas the secondary binding sites of *Sleeping Beauty* and *Bari*-like elements exist as tandem repeats within longer, bipartite TIRs (Ivics et al., 1997; Moschetti et al., 2008; Rio, 2002). A similar structure has been found in *Foldback* and *Phantom* elements, although whether their tandem repeats act as binding sites remains untested (Cheng et al., 2000; Marquez and Pritham, 2010). Despite having a transposase related to the *P-element*, the *Galileo* TIRs share much in common with the *Foldback* elements. At more than 1 kb long, the functionality of these repeats remains uncertain. At first sight they might seem to be counterproductive as transpositional efficiency is usually negatively correlated with the length of the transposon (Atkinson and Chalmers, 2010). The presence of multiple transposase binding sites may somehow offset the penalty associated with increased length of the transposon. In fact, this strategy has been used to improve the efficiency of artificial transposon (Zayed et al., 2004).

## 4 Conclusions

This work constitutes a first step in the characterization of the *Galileo* transposition. Although we did not detect *in vivo* transposition with a reconstructed consensus sequence, we detected specific binding by the N-terminal THAP domain of the transposase. We located the specific binding site about 70 bp from the transposon end, together with two additional binding sites within the unusually long and internally repetitive TIRs. This is the first demonstration of the functional significance of extremely long TIRs observed in members of the *Galileo* and *Foldback* transposon families.

## 5 Methods

### 5.1 Amplification of *D. buzzatii Galileo* transposase coding sequence by PCR

Three overlapping regions that span the entire *Galileo* transposase were PCR amplified from *D. buzzatii* strains st-1, Maz-4, j-9, jq7-4, jz3-2, jq7-1, Sar-9 and j-4. Reactions were performed in a total volume of 25 μl and contained 100–200 ng of genomic DNA, 20 pmol of each primer, 200 μM dNTPs, 1.5 mM MgCl_2_ and 1–1.5 units of Taq DNA polymerase. The products were gel-purified and sequenced. Primer sequences are listed in Table S2.

### 5.2 Generation of THAP domain sequences

A consensus sequence of the Dbuz\*Galileo* transposase segment was generated with the PCR products using the majority rule (Geneious assembly algorithm in Geneious (Drummond et al., 2010)). This consensus sequence differs from the reported Dbuz\*Galileo* sequence (Marzo et al., 2008) by 5 nucleotides and can be translated into a fully functional protein. The THAP domain region of the consensus sequence is located in the N-terminal 450 bp portion.

Consensus sequences were also generated for *D. ananassae* and *D. mojavensis* transposase sequences reported previously (Marzo et al., 2008). The chosen sequences are listed in the Supplementary Table S1. They were aligned with the MUSCLE 4.8.4 algorithm (Edgar, 2004) implemented in the Geneious software (Drummond et al., 2010) and a majority rule consensus of the THAP domain was generated (450 bp). As described previously, there are four different *Galileo* subfamilies (C–F) in *D. mojavensis* (Marzo et al., 2008). Here we generated transposase consensus sequences for the *Galileo*C and *Galileo*D subfamilies.

Finally, a reconstruction of the 450 bp ancestral THAP domain coding sequences was carried out for *D. ananassae* and *D. mojavensis* (C and D subfamilies). MUSCLE 4.8.4 (Edgar, 2004) alignments were used for generating the best trees by maximum likelihood using RAxML phylogenetic software (GTR + gamma evolution model) (Stamatakis, 2006). The trees were rooted with an appropriate outgroup using the FigTree 1.3.1 (Rambaut, 2006) program and, after rooting, the outgroup was removed from the tree manually. These rooted phylogenetic trees and the alignments were used for inferring the ancestral sequence by maximum likelihood using the CODEML binary from PAML software (Yang, 1997) (parameters: seqtype = 1 (codons); codonfreq = 2; NSsites = 0 1; rateancestor = 1; fix_blength = 1).

### 5.3 TIR cloning and secondary binding site sequences

In order to test the DNA binding ability of the *Galileo* THAP domains, a 150 bp TIR consensus sequence was generated for *Galileo* elements in *D. buzzatii* (*Galileo*G, *Galileo*N and *Galileo*K subfamilies), *D. mojavensis* (*Galileo*C and *Galileo*D subfamilies) and *D. ananassae*. These consensus sequences were generated using the majority rule, as described above. Gene synthesis was used to create plasmid pRC1525, which contained the concatenated inferred sequences of *Galileo* TIRs with representative target site duplications. Unique restriction sites were located in between each TIR so that they could be released individually from the vector. Fragments were labeled using 32P dCTP using an exo- Klenow polymerase. The secondary binding sites, BS2 and BS3, were synthesized as 50 bp oligonucleotides, annealed, labeled using T4 polynucleotide kinase and gel purified with standard protocols.

### 5.4 THAP protein expression

The inferred ancestral and consensus 450 bp sequences were codon optimized and synthesized. From these sequences a 270 bp (90 amino acid) predicted core THAP domain was PCR amplified (Phusion enzyme) and cloned in pOPINM (N-terminal MBP-tag vector from The Oxford Protein Production Facility, UK) using the In-Fusion cloning technology (Clontech Inc.). Since no ancestral sequence was reconstructed for the *D. buzzatii* domain, the 450 bp THAP sequence (150 amino acid) was cloned directly in the pOPINM expression vector. The expression vectors with the THAP domains were sequenced to confirm the ORF and transformed in BL21 (DE3) *E. coli* for protein expression. The LB medium was supplemented with 100 μM of ZnCl_2_. Expression was induced with 1 mM IPTG when the LB culture reached OD_600_ = 0.5 and grown overnight at 16 °C. The cells were harvested by centrifugation and resuspended in HSG buffer, which contained 50 mM HEPES pH 7.5, 200 mM NaCl, 2 mM dithiothreitol, 5 mM EDTA and 10% glycerol. The cells were lysed in a French press and centrifuged at 25,000 g for 30 min. The supernatant was loaded onto an amylose resin column (New England Biolabs). The column was washed several times with HSG buffer and the protein eluted with HSG buffer plus 10 mM maltose. The fractions containing MBP-THAP domain were pooled and aliquots were stored at − 80 °C.

### 5.5 Electrophoresis mobility shift assay (EMSA)

Purified recombinant THAP domains were incubated for 2 h at room temperature with the labeled TIR in 20 μl reaction of binding buffer containing 20 mM Tris–HCl, pH 7.5, 100 mM KCl, 100 μg/ml bovine serum albumin, 2.5 mM DTT, and 5% glycerol. The reactions were loaded in a 4% TAE-polyacrylamide gel and electrophoresed for 2 h at 300 V at 4 °C.

### 5.6 Footprint assay

A sample of the binding reaction mixture was digested by 0.05 U of DNase I for 1 min at room temperature. The enzyme was diluted to 1 U/μl with dilution buffer (5 mM MgCl_2_, 0.5 mM CaCl_2_). The reaction was stopped using 1 μl of 500 mM EDTA. The complexes were separated using the EMSA. The wet gel was exposed to X-ray film to locate the complexes, which were excised. The gel slice was incubated in TE buffer plus 100 mM NaCl overnight to allow the DNA to diffuse into the solution. The solution was extracted with phenol–chloroform and the DNA was recovered by ethanol precipitation. The cleavage pattern was analyzed by electrophoresis on a 5% polyacrylamide sequencing gel. DMS/piperidine reactions were performed following standard procedures to reveal G positions and were used to localize the DNase I protected regions.

### 5.7 *In vivo Galileo* transposition experiment

The helper plasmid pTURBO-*Galileo* (pRC1510) encoding the inferred Dbuz\*Galileo* consensus transposase ORF was generated by PCR (primer sequences are listed in Table S2). The PCR fragments were assembled using the unique silent restriction sites at each end of the fragments. This consensus ORF was cloned in the pTURBO plasmid replacing the *P-element* transposase (pUChsΔ2-3, FlyBase recombinant construct FBmc0000938, pRC1501). For this purpose, a PCR of whole pTURBO sequence except the *P-element* ORF was performed and two unique restriction sites (MluI and EagI) were added for cloning the *Galileo* transposase. After cloning the ORF was sequenced.

The donor plasmid, pCASPER-*Galileo* (pRC1517) was based on pCaSpeR-4 (FlyBase recombinant construct FBmc0000178 (pRC1502)). Two PCRs were performed for amplifying and ligating all the plasmid without the *P-element* sequences. In this step 4 unique restriction sites were added (PstI, NotI, NsiI and BamHI) surrounding the mini *white* gene. These 4 unique restriction sites were used for cloning the consensus 150-pb *Galileo* TIR in the inverted repeat configuration on either side of the mini*white* gene (TIR1: PstI and NotI, TIR2: NsiI and BamHI). The mini*white* ORF and the TIR were sequenced. The PCRs carried out in this section were performed with Phusion polymerase (Finnzymes).

#### 5.7.1 *Drosophila* injections

3 different injections were performed in *Drosophila melanogaster white* embryos (strain w1118, Genetic Services Inc. USA): one with the *P-element* plasmids without any change as a positive control (pRC1501 — helper and pRC1502 — donor), another with the two *Galileo* generated plasmids (pRC1510 — helper and pRC1517 — donor) and a last one with only the plasmid pRC1517 as a negative control. The injected adults (91 positive controls, 99 *Galileo* transposition elements and 96 negative controls) were each crossed with 3 virgin females or 3 males depending on the sex of the injected fly. The tubes of the crosses with *Drosophila* media were changed every two days (in the case of one injected male with 3 virgin females) or every 4 days (in the case of one injected female with 3 males) during 12 days. Finally, the F1 offspring of each cross was counted and non-*white* eyes were screened (from light orange to deep red eyes) as a marker of transposition activity.

The following are the supplementary data related to this article.

Fig. S1 DNase footprinting gel.
The entire gel image from Fig. 5A is shown.

Table S1 Sequences used for inferring the THAP domain sequences: (CAF1 assemblies).

Table S2 Primers used in this work.

## Conflict of interest

The authors declare that they have no competing interests.

## Figures and Tables

**Table 1:** *Drosophila* melanogaster embryo injections.

	*Pnnelement* Positive control	*Galileo* experiment	*Galileo* Negative control
Surviving adults crossed	91	99	96
Crosses yielding red eyed F1s	19	0	0
Total number of F1s screened	27021	32537	31201
Total number of red eyed individuals	384	0	0
