# Supplementary figures and images for: Identification of multiple binding sites for the THAP domain of the Galileo transposase in the long terminal inverted-repeats
Source: Gene. 2013 Aug 1;525(1):84–91. doi: 10.1016/j.gene.2013.04.050 (PMC3688188; doi:10.1016/j.gene.2013.04.050)

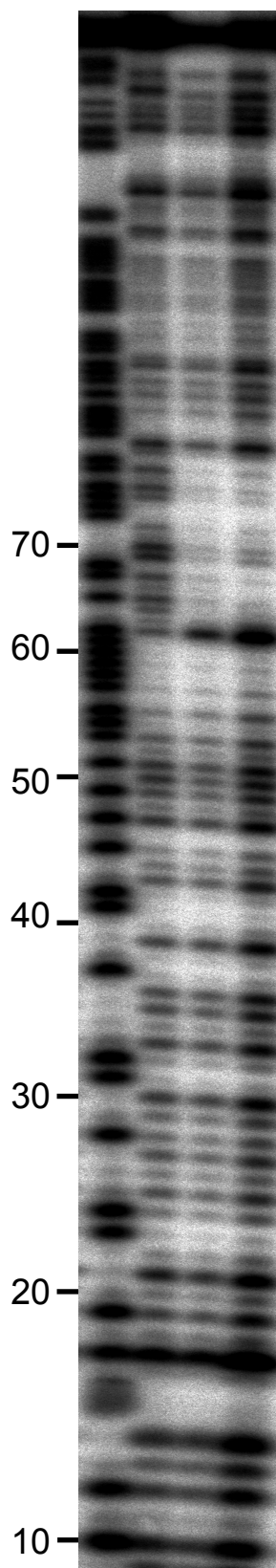

Supplement: Fig. S1 — DNase footprinting gel. The entire gel image from Fig. 5A is shown. [file mmc1.pdf]
