# Supplementary material for: Identification of multiple binding sites for the THAP domain of the Galileo transposase in the long terminal inverted-repeats
Source: Gene. 2013 Aug 1;525(1):84–91. doi: 10.1016/j.gene.2013.04.050 (PMC3688188; doi:10.1016/j.gene.2013.04.050)
Supplement: Table S1 — Sequences used for inferring the THAP domain sequences: (CAF1 assemblies). [file mmc2.doc]

Table S1

Sequences used for inferring the THAP domain sequences: (CAF1 assemblies)

| Species/Subfamily |  | Coordinates |
| --- | --- | --- |
| *D. mojavensis* C | scaffold_6262 | 13889-19752 |
|  | scaffold_6541 | 1141978-1149130 |
|  | scaffold_6500 | 31288762-312953303 |
|  | scaffold_6358 | 1-5345 |
|  | scaffold_6500 | 31981325-31980812 |
| *D. mojavensis D* | scaffold_6500 | 31458921-31464785 |
|  | scaffold_6482 | 614003-617184 |
|  | scaffold_6482 | 617185-618411 |
|  | scaffold_6485 | 39163-45738 |
|  | scaffold_6540 | 1175880-1182997 |
| *D. ananassae* | contig_15979 | 71824-74395 |
|  | contig_11169 | 1-2142 |
|  | contig_19410 | 7756-12565 |
|  | scaffold_13082 | 2449985-2467038 |
