# Supplementary material for: Identification of multiple binding sites for the THAP domain of the Galileo transposase in the long terminal inverted-repeats
Source: Gene. 2013 Aug 1;525(1):84–91. doi: 10.1016/j.gene.2013.04.050 (PMC3688188; doi:10.1016/j.gene.2013.04.050)
Supplement: Table S2 — Primers used in this work. [file mmc3.doc]

Table S2

Primers used in this work

| Name | Sequence (5’-3’) | Template |
| --- | --- | --- |
| TIR1_PstI-TSD-F | GACAGTCTGCAGGTGATAGCACTAACCATACAACACATAGACTG | pGPE_Dbuz\Galileo TIR |
| TIR1_NotI_150bp-R | GTGACTGAGCGGCCGCCGGAATGATTTTGTCATCA | pGPE_Dbuz\Galileo TIR |
| TIR2_BamHI-TSD-R | CTATGTGGATCCGTGATAGCACTAACCATACAACACATAGACTG | pGPE_Dbuz\Galileo TIR |
| TIR2_NsiI_150bp-F | GTGACTGAATGCATCGGAATGATTTTGTCATCA | pGPE_Dbuz\Galileo TIR |
| Dbuz_TPase_EagI_Met-F | GATCTACGGCCGAAAATGGCGCAAATAAGTGTTGTG | Consensus TPase |
| Dbuz_TPase_end-MluI-R | GACGAAACGCGTTATTTTTATTCACGAATCATTTTCAGTTTACTTTTAC | Consensus TPase |
| pTURBO-EagI-R | CTAGATCGGCCGTTTATTCCACGTAAGGGTTAATG | pTURBO |
| pTURBO-MluI-F | CTTCGTACGCGTGAGTTAATTCAAACCCCACG | pTURBO |
| Bb_pCAS-BamHI-F | TCTGATGGATCCGCAAGGAGTAGCCGACATATATC | pCASPER |
| Bb_pCAS-PstI-R | TAAGCATCTGCAGCGGAGAAGTTAAGCGTCTC | pCASPER |
| White-NsiI-BamHI-R | CATGCTAGGATCCATAGCTAGTTGAGATGCATCTACACAAGGAAC | pCASPER |
| White-PstI-(NotI)-F | CATGCTCTGCAGACTAGTGGCCTATGCGGC | pCASPER |
| GalBspEI-R | TTCATCCGGAATACAATTTCCAGATATTGAAG | Previous TPase sequence |
| GalBspEI-F | GTATTCCGGATGAAGATTCAATGCTAG | Previous TPase sequence |
| GalBsaI-F | TCTACGGGTCTCATGAGGTTAAAATTAAGAAAGGTCTTC | Previous TPase sequence |
| GalMet-F | ATGGCGCAAATAAGTGTTGTGAACG | Previous TPase sequence |
| GalBsaI-R | TCTTAAGGTCTCCCTCATCGAAAACTAATACTGCATAC | Previous TPase sequence |
| GalStop-R | TTATTCACGAATCATTTTCAGTTTACTTTTAC | Previous TPase sequence |
| An_pM-R | ATGGTCTAGAAAGCTTTAGTTCGGACACAGCAGGGAGT | BioS&T plasmid |
| AnA-pM-F | AAGTTCTGTTTCAGGGCCCGATGAATCGCCAGAACATCCG | BioS&T plasmid |
| AnC-pM-F | AAGTTCTGTTTCAGGGCCCGATGAATCGCCAGAACGTTCG | BioS&T plasmid |
| Bu-150pM-F | AAGTTCTGTTTCAGGGCCCGATGGCTCAGATCAGCGTGG | BioS&T plasmid |
| Bu-150-pM-R | ATGGTCTAGAAAGCTTTAAAAAATCAGCAGGTTTTCAATCAG | BioS&T plasmid |
| Bu_pM-F | AAGTTCTGTTTCAGGGCCCGCGTAAATCCGGTGCGAAATG | BioS&T plasmid |
| Bu_pM-R | ATGGTCTAGAAAGCTTTAATTGGAAAAGAAGTTCGGATCG | BioS&T plasmid |
| MoC_pM-F | AAGTTCTGTTTCAGGGCCCGCAGCGTAATGGCGGTAAGTG | BioS&T plasmid |
| MoC_pM-R | ATGGTCTAGAAAGCTTTAGTTGTTAGAAATCAGGTTGGAGTTACC | BioS&T plasmid |
| MoD-pM-F | AAGTTCTGTTTCAGGGCCCGCGTCGTAACGGTGGTAAATGC | BioS&T plasmid |
| MoD-pM-R | ATGGTCTAGAAAGCTTTAATTGTTGGACAGCAGGTTGC | BioS&T plasmid |
